# Application and Analysis of Bipolar Membrane Electrodialysis for LiOH Production at High Electrolyte Concentrations: Current Scope and Challenges

**DOI:** 10.3390/membranes11080575

**Published:** 2021-07-29

**Authors:** Alonso González, Mario Grágeda, Adrián Quispe, Svetlana Ushak, Philippe Sistat, Marc Cretin

**Affiliations:** 1 Departamento de Ingeniería Química y Procesos de Minerales and Center for Advanced Study of Lithium and Industrial Minerals (CELiMIN), Universidad de Antofagasta, Campus Coloso, Av Universidad de Antofagasta, Antofagasta 02800, Chile; alonso.gonzalez@celimin.com (A.G.); adrian.quispe.huayta@ua.cl (A.Q.); svetlana.ushak@uantof.cl (S.U.); 2 Institut Européen des Membranes, IEM, UMR-5635, Université de Montpellier, ENSCM, CNRS, Place Eugène Bataillon, CEDEX 5, 34095 Montpellier, France; philippe.sistat@umontpellier.fr (P.S.); marc.cretin@umontpellier.fr (M.C.)

**Keywords:** lithium hydroxide, bipolar membrane electrodialysis, high concentration, lithium brine, sustainable process

## Abstract

The objective of this work was to evaluate obtaining LiOH directly from brines with high LiCl concentrations using bipolar membrane electrodialysis by the analysis of Li^+^ ion transport phenomena. For this purpose, Neosepta BP and Fumasep FBM bipolar membranes were characterized by linear sweep voltammetry, and the Li^+^ transport number in cation-exchange membranes was determined. In addition, a laboratory-scale reactor was designed, constructed, and tested to develop experimental LiOH production tests. The selected LiCl concentration range, based on productive process concentrations for Salar de Atacama (Chile), was between 14 and 34 wt%. Concentration and current density effects on LiOH production, current efficiency, and specific electricity consumption were evaluated. The highest current efficiency obtained was 0.77 at initial concentrations of LiOH 0.5 wt% and LiCl 14 wt%. On the other hand, a concentrated LiOH solution (between 3.34 wt% and 4.35 wt%, with a solution purity between 96.0% and 95.4%, respectively) was obtained. The results of this work show the feasibility of LiOH production from concentrated brines by means of bipolar membrane electrodialysis, bringing the implementation of this technology closer to LiOH production on a larger scale. Moreover, being an electrochemical process, this could be driven by Solar PV, taking advantage of the high solar radiation conditions in the Atacama Desert in Chile.

## 1. Introduction

In recent years, lithium has become a mineral of great interest worldwide. Its demand has increased due to its use in lithium-ion batteries for electric vehicles and consumer electronics, which has been boosted in some countries by energy policies that promote clean energy usage. Currently, lithium hydroxide (LiOH) shows high projections in the production of battery cathodes [1,2]. By 2030, lithium consumption in electric vehicle batteries is expected to account for 80% of aggregate lithium consumption. By the same year, lithium hydroxide is projected to account for 57% of lithium compound demand, compared to 24% in 2019 [3]. Some key advantages of cathodes produced from lithium hydroxide over other chemical compounds are better power density, longer life cycle, and improved safety characteristics [4,5,6,7]. 

Conventional industrial processes for obtaining lithium salts from brines include several stages and unit operations. Lithium is extracted by pumping natural brines to evaporation ponds, where solar radiation produces energy to evaporate brine water, precipitating salts in a sequential process lasting between 12 and 15 months, until a concentration of 5.5–6.0 wt% lithium is reached [8]. Lithium-concentrated brine is then used as a raw material to obtain lithium carbonate through solvent extraction, purification, chemical reaction, filtration, and classification processes [9]. Some of the lithium carbonate obtained is technical grade (99.0% purity), and is subsequently used as a raw material to obtain lithium hydroxide by the reaction ${\mathrm{Li}}_{2}{\mathrm{CO}}_{3}+\mathrm{Ca}{\left(\mathrm{OH}\right)}_{2}\to 2\mathrm{LiOH}+{\mathrm{CaCO}}_{3}$. As a product of this reaction, an aqueous solution is obtained that reaches approximately 3 wt% LiOH concentration, which is subjected to an evaporation and crystallization process to obtain lithium hydroxide monohydrate. At the same time, calcium carbonate is obtained as waste, which is separated by settling tanks and filters. Conventional processing from concentrated brine to lithium hydroxide monohydrate crystals requires several pieces of equipment and, therefore, a large installation space, as well as the addition of chemical reagents (e.g., Na_2_CO_3_, CaO, among others), producing liquid waste (mother liquor) and solid wastes (e.g., Mg(OH)_2_, CaCO_3_, among others) [10]. 

Ion-exchange-membrane-based electrochemical processes are an alternative for the production of alkaline products, avoiding the use of chemical reagents and reducing waste production. Thais is the case of the chloralkali process for NaOH production. In this process, a cationic membrane is used between two electrodes and, by applying an electric current, water reduction at the cathode is achieved to generate NaOH and H_2_ gas through the semi-reaction $2H_{2}O+2e^{-}\to H_{2}+2{\mathrm{OH}}^{-}$. Gas generation is an electrochemical phenomenon that is best avoided for certain applications where it is not used and simply discarded. This can be achieved by using bipolar membranes, which are also less expensive compared to an electrode for OH^−^ ion generation. A bipolar membrane (BPM) consists of a cationic layer, an anionic layer, and an intermediate zone that allows water catalysis of dissociation into OH^−^ and H^+^ ions without gas generation [11,12]. Thus, production of acids and bases from a salt solution is possible. Bipolar membrane electrodialysis (BMED) is a technology that has been studied in recent years for various applications, such as desalination [13,14,15,16] and sodium hydroxide production [17,18,19], as well as boron recovery [20] and acid recovery [21,22]. 

In the case of lithium, its recovery from waste streams has been studied using conventional electrodialysis [23] and bipolar membrane electrodialysis [21,24,25,26], generally recovering lithium in dilute LiOH solutions. Few works have been reported whose main focus is the production of lithium hydroxide by electromembrane processes [27]. They usually study membranes’ interaction with aqueous solutions more dilute than those used in the lithium hydroxide production industry. Among them, the work of Melnikov et al. [28] stands out, where from a LiCl solution with organic solvents, the authors produced LiOH by bipolar membrane electrodialysis, reaching a LiOH concentration of 0.3 M with a specific energy consumption of 6.6 kWh∙kg^−1^ and a current efficiency of 0.6. Grageda et al. [29] analyzed a membrane electrodialysis process to produce high-purity lithium hydroxide, determining the effects of concentration, current density, and temperature on process energy performance and product purity; the authors reported a specific energy consumption of 7.25 kWh∙kg^−1^ of LiOH. On the other hand, Ryabtsev et al. [30] used membrane electrolysis to obtain lithium hydroxide from lithium carbonate treated with sulfuric acid, taking advantage of the high solubility of Li_2_SO_4_, and managing to obtain a high average LiOH concentration of 45 g∙L^−1^, similar to that obtained in the conventional LiOH process. Another interesting work is the one carried out by Jiang et al. [31], in which LiOH with a purity of 95% was obtained from aqueous solutions of Li_2_CO_3_ (0.18 M), with a specific electricity consumption of 6.66 kWh∙kg^−1^. BMED has also been studied for hydrochloric acid production, reporting specific energy consumptions between 4.4 kWh∙kg^−1^ and 8.3 kWh∙kg^−1^ of HCl [32,33]. 

In recent years, interest in the application of bipolar membranes has increased due to their technical, environmental, and economic advantages for the production of acids and bases, compared to their conventional production processes [27]. Today, this technology is applied for research and some pilot projects [34,35]. Electrodialysis processes are affected by different variables, such as current density, electrolyte concentration, flow rate, osmotic pressure, and ion activity. These parameters affect electrochemical equilibrium between membrane-separated solutions by influencing ion transport across the membrane. During a concentration process with ion-exchange membranes, transport rate varies according to changes in concentration and membrane stability. On the other hand, co-ion flux, osmosis, and electro-osmosis affect process performance. For the application of LiOH in lithium battery materials, high purity is required, which to date has not been achieved using BMED. Therefore, there is a need to understand the transport mechanism of Li^+^ ions across membranes. This work analyzes the feasibility of BMED for application in lithium hydroxide production from concentrated brines, with the aim of achieving high-purity lithium hydroxide concentrations for applications in the battery industry. Thus, our aim is to reduce the energy consumption of the overall production process by achieving a smaller gap between final LiOH concentration and saturation concentration, reducing energy expenditure in the evaporation, crystallization, and lithium hydroxide production stages that currently exist. This work presents the results of the feasibility and scope of LiOH production by BMED using high concentrations, identifying the main phenomena affecting lithium transport and LiOH formation throughout the concentration process with BMED. The main novelty of this study is the use of high-concentrated aqueous solutions, similar to those obtained during the industrial lithium concentration process in the Salar de Atacama. Moreover, experimental process performance data are presented, establishing the current scope of BMED technology in industrial LiOH production. In addition, the energy supply for this process could be photovoltaic solar energy [33], given the high solar irradiation levels in the Salar de Atacama, making this an even more sustainable process from energy point of view.

## 2. Materials and Methods

### 2.1. Materials

Cationic (Neosepta CMX and CMB) and anionic (Neosepta AMX, manufactured by Astom Corporation, Tokyo, Japan) ion-exchange membranes were used in this work. The bipolar membranes used were Neosepta BP (Astom Corporation, Tokyo, Japan) and Fumasep FBM (FuMA-Tech GmbH, Bietigheim-Bissingen, Germany). The technical specifications of the membranes are shown in 

Table 1 and Table 2. For the preparation of aqueous solutions, LiCl (Winkler ltda, Santiago, Chile), LiOH, HCl, Na_2_SO_4_ (Merck, Darmstadt, Germany), and deionized water were used as reagents. The presence of other elements such as K, Mg, and Ca present in Salar de Atacama brine was not considered, as this study focuses on transport phenomena associated with lithium and the effect of high concentrations. Therefore, a purified brine free of Mg and Ca—as can be obtained by chemical precipitation and ion-exchange processes—was considered for experimental development [36]. The presence of these elements should be reduced to the minimum possible in order to avoid membrane poisoning [37].

### 2.2. Measurement of Water Uptake and Thickness of Membranes

In order to ascertain the influence of different operating solutions on membranes’ water uptake and thickness, these were equilibrated in their respective operating solutions. Thus, for cation-exchange membranes, LiOH (0.5–5.0 wt%) and LiCl (14–34 wt%) solutions were used, while for bipolar membranes, LiOH and HCl (0.5–5.0 wt%) solutions were used, in which membranes were equilibrated for at least 24 h. Then, the electrolytes on the membranes’ surface were carefully cleaned, thickness was measured using a high-accuracy digital thickness gauge, and the wet membranes’ mass was recorded. Subsequently, membranes were dried in an oven at 70 °C until they showed zero mass variation. Water uptake (W_u_) was calculated using Equation (1), from dry mass ($W_{d}$) and wet mass ($W_{w}$) values, in grams. At least two replicates were performed for each measurement.

(1) $$W_{u}=\frac{W_{w}-W_{d}}{W_{d}}\times 100$$

### 2.3. Determination of Lithium Transport Number

**Figure 1:**
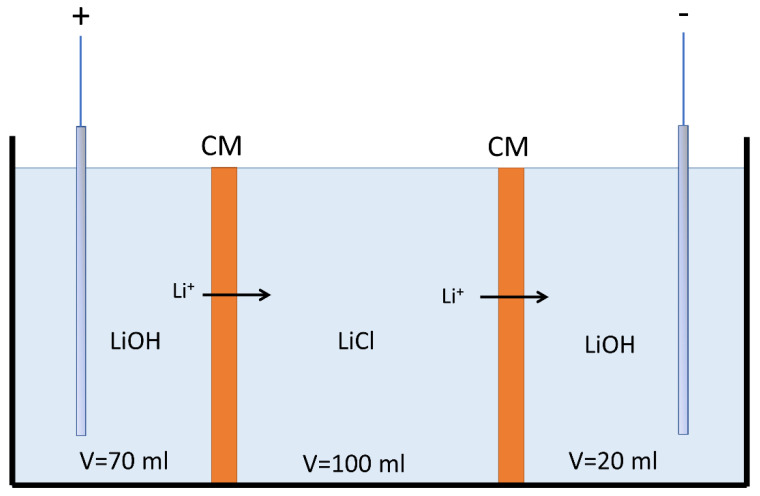
Hittorf method experimental scheme for the determination of lithium ion transport number (CM: Cation exchange membrane).

In order to determine the influence of current density and initial lithium concentration on transport in cation-exchange membranes, tests were performed to determine the lithium transport number. For this purpose, the Hittorf method was used according to the scheme in Figure 1, in which a three-compartment cell separated by two cation-exchange membranes with an effective membrane area of 7 cm^2^ was used. Two different cation-exchange membranes were used: Neosepta CMX and CMB. In the cathodic compartment, lithium from a LiCl solution reacted with OH^−^ anions and was concentrated in LiOH form within 60 min. To reduce the concentration variation of LiCl solution, a larger volume of LiCl (100 mL) was used compared to the LiOH solution receiving lithium ions (20 mL). A 2.5 wt% LiOH solution (g/100 g solution) was used as an anolyte under the assumption that lithium ions are transported to the LiCl compartment at the same rate at which they migrate to the cathode, contributing to keep its concentration unchanged. After 60 min, the LiOH solution in the cathode compartment was extracted and analyzed by ion chromatography to determine the difference in Li concentration and calculate its Li ion molar flux ($J_{\mathrm{Li}}$) across the cation-exchange membrane using Equation (2):

(2) $$J_{\mathrm{Li}}=\frac{C_{\mathrm{Li},0}\cdot V_{\mathrm{LiOH},0}-C_{\mathrm{Li},f}\cdot V_{\mathrm{LiOH},f}}{A\cdot t}$$

where $C_{\mathrm{Li}}$ is the molar concentration of lithium in the LiOH solution (mol∙L^−1^), $V_{\mathrm{LiOH}}$ is the volume of the LiOH solution (L), A is the effective membrane area (m^2^), and t is the time (s). Subscripts 0 and f refer to initial and final time, respectively. 

Once molar flux ($J_{\mathrm{Li}}$) was determined, the lithium ion transport number $t_{\mathrm{Li}}$ was calculated according to Equation (3):

(3) $$t_{\mathrm{Li}}=\frac{F\cdot z_{\mathrm{Li}}\cdot J_{\mathrm{Li}}}{I}$$

where F is the Faraday constant ($96,485\;A\cdot s\cdot {\mathrm{mol}}^{-1}$), $z_{\mathrm{Li}}$ is the lithium-ion valence ($z_{\mathrm{Li}}=1$), $J_{\mathrm{Li}}$ is the lithium-ion molar flux ($\mathrm{mol}\cdot m^{-2}\cdot s^{-1}$), and I is the electric current (A).

The different concentration conditions used are presented in Table 3. The effects of current densities (300, 700, and 1100 A∙m^−2^) were also studied. All tests were performed at room temperature (20–22 °C). The pH of the LiCl solution was measured before and after the experiment.

Electrolyte concentration selection corresponded to ranges established based on concentrated lithium brine obtained from solar evaporation ponds used in productive processes at the Salar de Atacama, where a brine rich in lithium up to 5.5–6.0 wt% was obtained, equivalent to approximately 33.6–36.6 wt% LiCl. In this work, concentrated LiCl solutions between 14 wt% and 34 wt% were considered. On the other hand, we performed experiments with a LiOH concentration range between 0.5 wt% and 8.0 wt%, covering higher concentrations than those obtained industrially (approximately 3.0 wt%) without reaching LiOH solution saturation concentration.

### 2.4. Linear Sweep Voltammetry (LSV)

In the characterization of bipolar membranes, the linear sweep voltammetry (LSV) technique allows the determination of the degree of salt leakage that occurs through them, and of the limitations of water diffusion towards bipolar membrane reactive interface [44]. In the case of cation-exchange membranes, LSV can be used to determine the limiting current density caused by the polarization concentration effect at membrane surface. Both phenomena affect the efficiency of the electrodialysis process.

**Figure 2:**
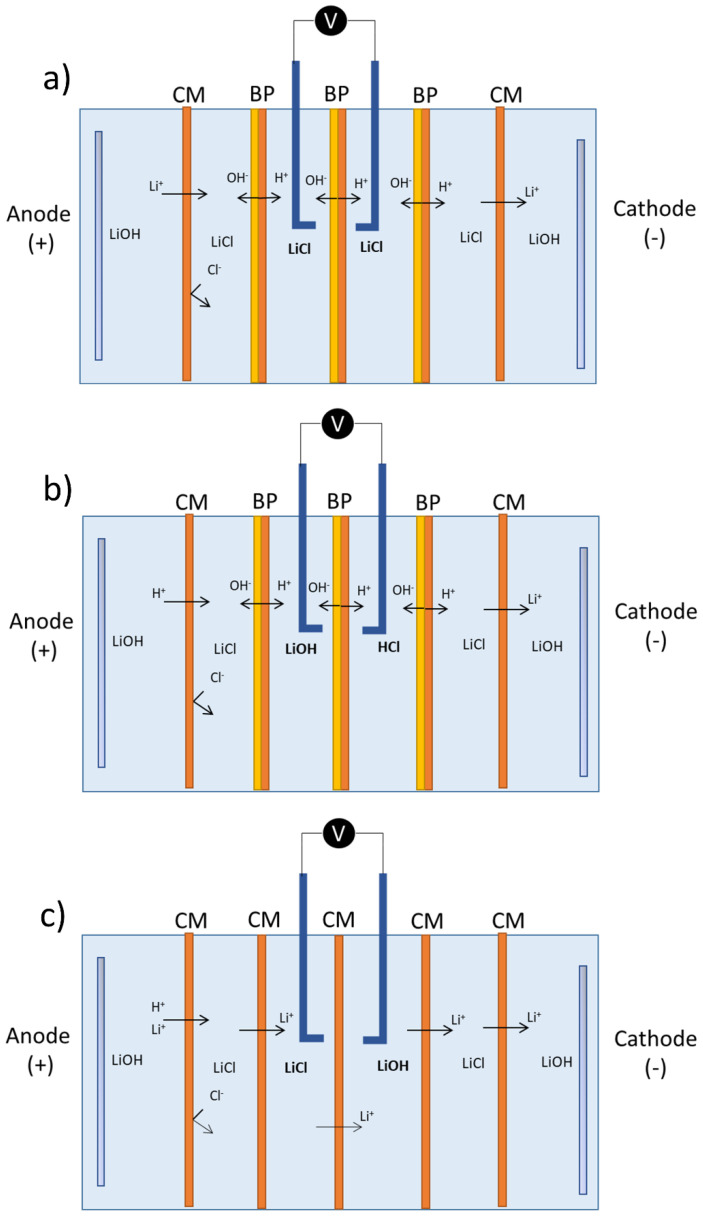
LSV tests’ cell configuration. (**a**) Limiting current density measurement as related to salt leakage in bipolar membranes (BP). (**b**) Bipolar membranes’ apparent electrical resistance measurements as related to operating conditions. (**c**) Cation-exchange membranes’ (CM) concentration polarization determination in the range under study.

To determine current–voltage curves and estimate membranes’ apparent electrical resistance, a six-compartment cell was used, according to electrochemical techniques indicated by Balster et al. [44]. To measure potential differences in the membranes under study, Haber–Luggin capillaries filled with 3 M KCl solution were used, where 99.99% silver wires connected to a voltmeter (Tenma 72-1016, Springboro, OH, USA) were placed. The experimental cell used had an effective membrane area of 4 cm^2^. The flow velocity in each compartment was between 1.0 and 1.4 cm∙s^−1^. The three cases considered are presented in Figure 2. The salt leakage and performance of bipolar membranes under production conditions were determined according to configurations (a) and (b), respectively. Cation-exchange membranes were tested according to configuration (c). The solution concentrations and membranes used in each test are presented in Table 4.

LSV tests were performed using a power supply (Tenma 72-2550, Springboro, OH 45066, USA); the step voltage increase was 0.15 V, and the corresponding current value was measured after 30–60 s after voltage stabilization was observed. For higher current values, more noise was observed in the measurements, so the step increase was 0.30 V with a waiting time between 60 and 90 s in order to reach voltage stabilization.

### 2.5. Bipolar Membrane Electrodialysis System

#### 2.5.1. Stack Design and Construction

**Figure 3:**
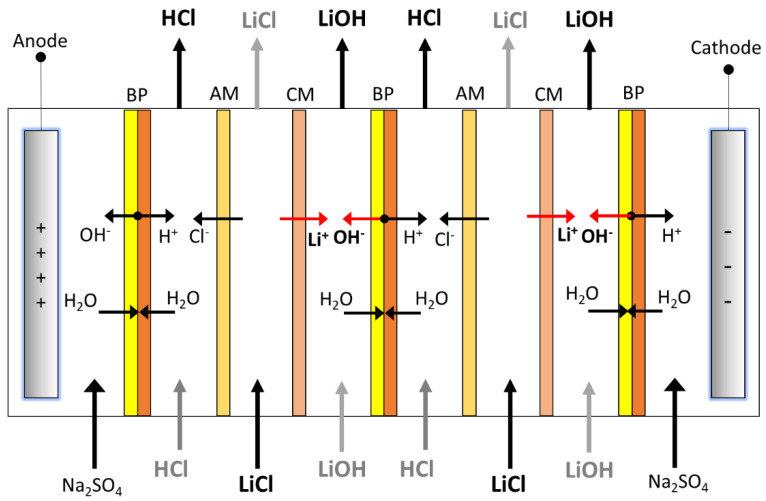
LiOH production by bipolar membrane electrodialysis; configuration of two three-compartment cells. BP: Bipolar membrane, AM: Anion exchange membrane, CM: Cation exchange membrane.

To perform experimental tests on LiOH production, an electrodialysis stack with bipolar membranes was designed and constructed. The stack was constructed from PTFE, with an effective membrane area of 27.5 cm^2^ (55 mm × 50 mm). The design was a “filter press” type where membranes and separators could be configured according to different flow compartments (see Figure 3). The separators were made of EPDM, with a thickness of 1.6 mm.

The electrodialysis stack’s repetitive unit corresponded to a three-compartment cell, consisting of a HCl compartment, an anion-exchange membrane, a LiCl compartment, a cation-exchange membrane, and a LiOH compartment. These were stacked between two bipolar membranes. In this way, the stack could be configured with several three-compartment cells, among which different electrolytes were distributed. The stack was fed with five flow streams corresponding to LiCl, LiOH, HCl, and two electrode solution streams. The main feed to the stack was the LiCl solution, from which lithium ions migrated into the LiOH solution and chloride ions migrated into the HCl stream. As the electrode solution, 0.5 M Na_2_SO_4_ was used; its purpose was to provide conductivity in the electrode compartment, and simultaneously prevent chlorine gas (Cl_2_) formation in the anode compartment.

#### 2.5.2. LiOH Production Experimental Tests Using BMED

**Figure 4:**
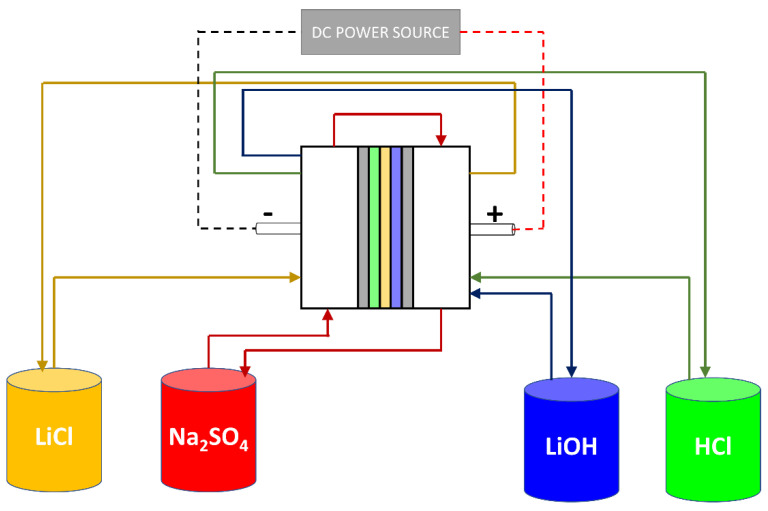
LiOH production system by BMED.

To test the LiOH production range, six long-running tests were performed. The system was configured in batch mode, recirculating different feed solutions and measuring concentration variation over time (see Figure 4). 

Flow rate in each compartment was set between 1.0 and 1.5 cm∙s^−1^ using peristaltic pumps (Watson-Marlow 520SN/R2, Falmouth, UK), and a DC power supply (GW Instek GPR-1810HD, New Taipei, Taiwan) was used to set the electric current.

Experimental long-running tests of LiOH production were performed according to the operating conditions shown in Table 5. Tests 1 and 2 compare the effects of two different cation-exchange membranes (CMX and CMB), while Tests 3 and 4 compare the effects of different bipolar membranes (Neosepta BP and Fumasep FBM). On the other hand, 14 wt% and 25 wt% LiCl concentration effects can be compared by Tests 1 and 3 at 1000 A∙m^−2^, while Tests 5 and 6 compare 14 wt% and 34 wt% LiCl concentrations at a current density of 500 A∙m^−2^. In the latter case, to obtain comparable initial and final LiOH concentration ranges, the number of compartments was increased to four three-compartment cells. Equal initial LiOH and HCl concentrations of 0.5 wt% were used in all tests. An initial LiCl concentration between 14 and 34 wt% was considered. Neosepta AMX was used as an anionic membrane. The cation-exchange membranes and bipolar membranes were previously conditioned in a 0.5 M LiOH solution for 24 h, while the anion-exchange membrane was conditioned in 0.5 M LiCl solution.

#### 2.5.3. SEC and ϕ Calculation

Concentrations of different elements—such as Li^+^, OH^−^, and Cl^−^—in the LiOH solution were measured at the end of each experiment. Lithium concentration was determined by atomic absorption spectrophotometry, chloride ion concentration was determined by an argentometric method, and hydroxide content was analyzed by acid–base volumetry.

For each test, specific electrical consumption (SEC) was calculated according to Equation (4):

(4) $$\mathrm{SEC}=\frac{I\cdot U\cdot t}{m}$$

where I is the electrical current (A), U is the average voltage (V), t is the process time (h), and m is the mass of LiOH produced (kg). On the other hand, current efficiency (ϕ) was calculated according to Equation (5):

(5) $$\phi =\frac{z\cdot F\cdot m}{N\cdot I\cdot t\cdot M}$$

where F is the Faraday constant ($96,485\;A\cdot s\cdot {\mathrm{mol}}^{-1}$), m is the mass of LiOH produced (g), z is the valence number, N is the number of LiOH compartments, I is the electrical current (A), t is the process time (s), and M is the molar mass.

Final LiOH solution purity was calculated according to Equation (6):

(6) $$P=\frac{m_{\mathrm{LiOH}}}{m_{\mathrm{TDS}}}\times 100$$

where $m_{\mathrm{LiOH}}$ is the LiOH mass in solution, and $m_{\mathrm{TDS}}$ is the mass of total dissolved salts (g).

## 3. Results

### 3.1. Water Uptake and Membrane Thickness

The CMX and CMB cation-exchange membranes’ water uptake and thickness were measured after equilibrium with LiCl and LiOH solutions, which they were in contact with during the BMED LiOH production process. Meanwhile, the same parameters were determined for the bipolar membranes Fumasep FBM and Neosepta BP in equilibrium with LiOH and HCl solutions at different concentrations. The results of water uptake and membrane thickness are presented in Table 6 and Table 7, respectively. In the cation-exchange membranes, water uptake in the membrane decreased with LiCl concentration. For a LiCl concentration of 34 wt%, the CMX membrane shows a 49.1% lower water uptake compared to a LiCl concentration of 14 wt%, while the CMB membrane shows a 38.3% lower water uptake for the same comparison. In contact with LiOH solutions, water uptake in the CMX membrane tended to decrease with concentration, while for the CMB membrane, water uptake increased for a 2.5 wt% LiOH solution and then decreased for a 5.0 wt% LiOH solution. The latter can be attributed to the high hydration shell associated with the lithium ion [45], which by increasing its concentration in the membrane increases water uptake in the membrane. Results suggest that between a concentration of 2.5 wt% and 5.0 wt%, water uptake would reach a maximum, which would then decrease with concentration due to osmotic deswelling [46]. Different behaviors observed in the CMX and CMB membranes can be attributed to the density of their polymeric structures [27]. In all cases, the CMB membrane presented a higher water uptake than the CMX membrane.

Bipolar membranes in contact with LiOH solutions presented a behavior similar to that observed for the CMB membrane. A higher water uptake was observed for a 2.5 wt% LiOH solution, which then decreased for a 5.0 wt% LiOH concentration. When they were equilibrated with HCl solutions, the same behavior was observed for the Fumasep FBM membrane as concentration increased, while the Neosepta BP membrane showed a slight tendency to decrease its water uptake. These differences would be determined by the ion-exchange layers’ specific characteristics in the respective membranes. The Fumasep FBM membrane showed a higher water uptake compared to the Neosepta BP membrane in all cases.

With respect to membrane thickness, the CMX membrane shows a clear tendency to decrease in thickness with concentration, which is consistent with the water uptake results. In contrast, the CMB membrane presents less variability in thickness for different LiOH concentrations, and an average thickness 1.2% lower for LiCl solutions. When comparing bipolar membranes, the Neosepta BP membrane shows a higher thickness than the Fumasep FBM membrane. Regarding different solutions, the Neosepta BP and Fumasep FBM membranes, when equilibrated with LiOH solutions, presented 15.4% and 10.4% higher thickness, respectively, compared to HCl solutions. This could be attributed to a greater lithium ion effect on the cation-exchange layer due to its higher hydrated radius compared to other monovalent cations [45].

### 3.2. Lithium Transport Number

**Figure 5:**
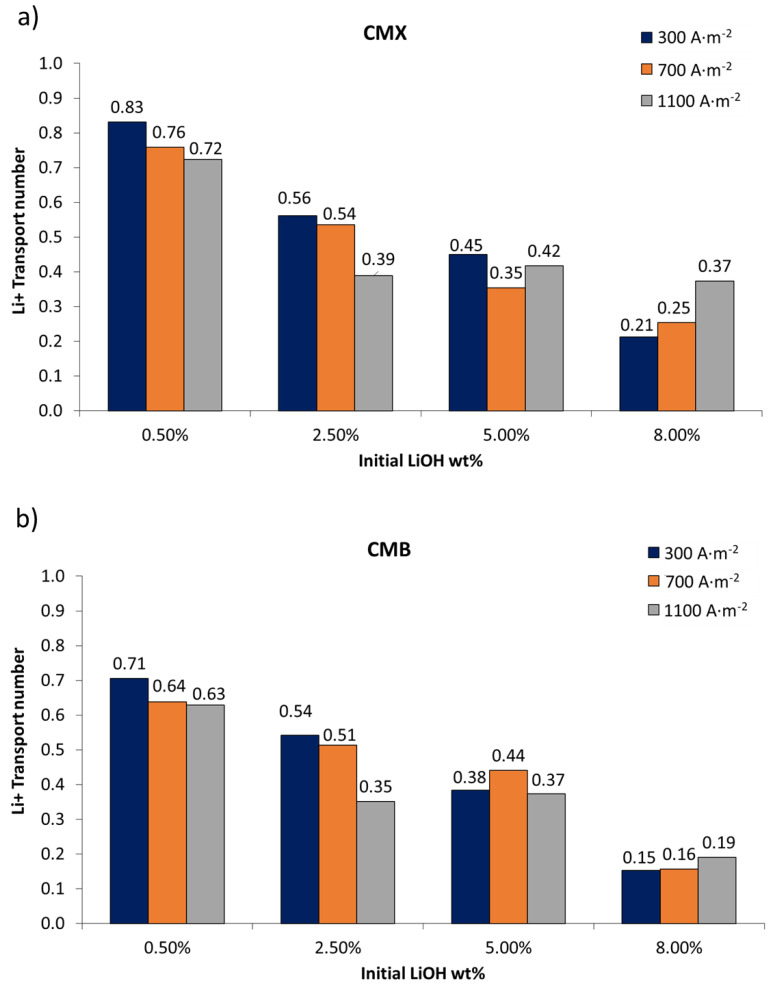
Lithium transport number in cation-exchange membranes according to LiOH concentration and current density (14 wt% LiCl): (**a**) CMX membrane; (**b**) CMB membrane.

The influence of current densities (300, 700, and 1100 A∙m^−2^) and initial LiOH concentrations (0.5, 2.5, 5.0, and 8.0 wt%) on lithium transport number at 14 wt% LiCl for CMX and CMB was studied; the results are summarized in Figure 5.

#### 3.2.1. Influence of Current Density

Current density shows influence on lithium transport. As can be seen in Figure 5, the lithium transport number decreases at higher current densities. This can be best observed for initial concentrations of 0.5 wt% and 2.5 wt% LiOH. In the CMX membrane, comparing LiOH concentrations at 0.5 wt% and 2.5 wt%, it was observed that when increasing current density from 300 to 1100 A∙m^−2^, the lithium transport number decreased by 13% and 30%, respectively. In the case of the CMB membrane for the same comparison, the lithium transport number decrease was 11% and 35%, respectively. At higher concentrations of LiOH (5.0 wt% and 8.0 wt%), although transport number variation followed the trend of decrease with LiOH concentration, it failed to clearly differentiate the effect of current density. However, at 1100 A∙m^−2^, it is appreciated that CMB membrane use showed a greater reduction in the lithium transport number—49% lower than that obtained with the CMX membrane. On the other hand, the results obtained for 8.0 wt% LiOH for both membranes (see Figure 5) indicate that a higher current density could counteract the effect of decreasing lithium transport number with concentration. This might be related to a lithium flux mostly associated to migration transport over diffusion transport, and could be explained qualitatively by the Nernst–Planck equation, where a higher electric field increases migration transport, while diffusive transport depends on the concentration difference on both sides of the membrane. Thus, high LiCl concentrations promote diffusive transport of lithium ions, whose effect on total transport decreases at higher current densities.

A problem observed when applying the Hittorf method at high concentrations was that when working with a 20-mL LiOH sample volume, the sensitivity of calculating molar flux and lithium-ion transport number increased due to the low final volume and high concentration, according to Equations (1) and (2).

#### 3.2.2. Influence of Initial LiOH Concentration

The lithium transport number decreases with LiOH concentration. For the CMX and CMB membranes, at 0.5 wt% LiOH concentration, the lithium transport number was in the range of 0.83–0.72 and 0.71–0.63, respectively. That is, more than 60% of the electric current was used for lithium transport through the cationic membranes. For the CMX membrane, when concentrating the LiOH solution from 0.5 wt% to 2.5, 5.0, and 8.0 wt%, the average decrease in the lithium transport number was 35%, 47%, and 64%, respectively. Meanwhile, in the CMB membrane, for the same concentrations, the average decrease was 29%, 40%, and 75%, respectively. The best result was 0.83 with the CMX membrane at an initial LiOH concentration of 0.5 wt%.

One of the possible causes of the decrease in the lithium transport number with LiOH concentration is the decrease in water content in the cationic membrane [37]. Due to the increase in concentration, membranes tend to lose water; this can be attributed to the accumulation of osmotic pressure within them [37,46]. According to the work varied out by Izquierdo-Gil et al. [47], comparing water content and salt transport in cationic Nafion membranes of different thickness, it was reported that water uptake increases with membrane thickness and decreases with cation size. In the present work, although the CMB membrane was thicker and showed higher water uptake than the CMX membrane, it had a lower lithium transport number—a condition that can be attributed to other characteristics of the CMB membrane, such as fixed charge density and polymeric matrix cross-linking [48]. Water uptake into the membrane allows ionic binding between two sides of the membrane, making electrical conductivity possible [47,49]. Increasing the concentration of electrolytes in contact with the membrane causes an increase in the concentration of counterions in the membrane, which in turn generates osmotic deswelling, further increasing its ion concentration [46]. Eventually, water loss and high counterion concentration can reduce the membrane’s fixed charge density. This has been explained thermodynamically by Kamcev et al. [46,50] by means of the counterion condensation model. Such a situation occurs when counterions close to fixed charges at a separation of less than the Bjerrum length do not have sufficient thermal energy to free themselves from the interaction of electrostatic forces, and tend to remain in this region, generating a shielding in fixed charges. Counterions trapped in this region are considered “condensed” and, therefore, immobile under a concentration gradient. However, counterions, under an electric field, exhibit higher diffusion coefficients than those that are not condensed, due to the shorter distance they must move in the membrane structure [51,52]. Although counterions’ condensation favors their transport, high concentration of these counterions implies a reduction in Donnan potential, and causes an increase in co-ion sorption in the membrane [52]. For the application of membranes in LiOH production with concentrated solutions, it is highly probable that counterion condensation occurs, which under an electric field would favor lithium migration through the cation-exchange membrane. However, this may increase OH^−^ co-ion concentration, which, due to its high mobility (the highest among all anions) promotes undesired OH^−^ transport, resulting in a decrease in the lithium transport number. The use of membranes with higher fixed charge density would present higher ionic conductivity, facilitating the transport of lithium ions across the membrane [51], as long as OH^−^ ion leakage is reduced. 

In this work, membranes’ water uptake tended to decrease with LiOH concentration. However, this variation does not capture the behavior of the lithium transport number for all cases. This lithium transport number reduction is better explained by the presence of OH^−^, and its interaction with the cation-exchange membrane. On the other hand, the decrease in the observed lithium transport number can be explained by the fact that the salt transport rate across the membrane decreases with decreasing concentration difference on both sides of the membrane, causing the permeation rate to decrease [47]—as occurs in the case of this work, where the LiOH concentration increased and the concentration difference with the LiCl solution decreased.

#### 3.2.3. pH Variation

During experiments, it was observed that the LiCl solution’s measured pH increased in all cases from pH 7–8 to a basic pH between 12–13. This increase can be attributed to the undesired migration of OH^−^ anions across the cation-exchange membrane due to the membrane not being 100% permselective, and also to the high OH^−^ ion mobility associated with the Grotthuss mechanism, involving proton hopping by ionization of water molecules. It has been reported that OH^−^ ions’ migration through the cation-exchange membranes reduces current efficiency in membrane processes for sodium hydroxide production [40], which in bipolar membrane electrodialysis can be manifested by an increase in the feed salt solution’s pH [53]. In this work, a higher pH increase was observed in the LiCl feed solution when working with more concentrated LiOH solutions. Hence, the lithium transport number decrease can be mainly attributed to electrical current transport by OH^−^ leakage through the cation-exchange membrane.

#### 3.2.4. Influence of LiCl Concentration 

**Figure 6:**
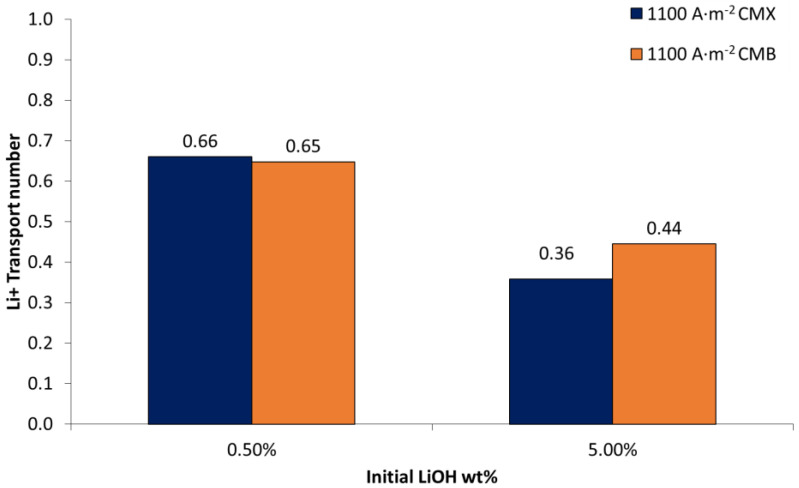
Lithium transport number in cation membranes (CMX and CMB) according to LiOH concentration and current density. LiCl 25 wt%.

To obtain a better understanding of the effects of LiCl concentration and concentration difference between electrolytes on each side of the membrane, additional experiments were performed for 0.5 wt% and 5.0 wt% LiOH at 25 wt% LiCl (Tests 5 and 6 in Table 3); The corresponding results are presented in Figure 6. When comparing the effects of LiCl concentration (see Figure 5 and Figure 6), results show that for the CMX membrane at 0.5 wt% LiOH concentration, the transport number decreased with LiCl concentration. Thus, when using a LiCl solution at 14 wt%, a value of 0.72 was obtained, while with a LiCl solution at 25 wt%, the transport number was 0.66. The same behavior was not obtained for the CMB membrane, where the transport number was 0.63–0.65 for the same comparison. This can be attributed to the fact that the CMB membrane has an average thickness 18.9% higher compared to the CMX membrane, with 50–60% higher exchange capacity and 13.3–37.5% higher water uptake (see Table 1, Table 6 and Table 7). In addition, the higher electrical resistance of the membrane would be associated with a denser polymeric structure [27]. These characteristics would allow the reduction of the influence of high LiCl concentrations on the lithium transport number. At higher LiOH concentrations, for the CMX membrane it was measured that at a concentration of 5.0 wt% LiOH, the lithium transport number decreased from 0.42 to 0.36 when using LiCl solutions of 14 wt% and 25 wt%, respectively. In the case of the CMB membrane, the lithium transport number was 0.37 and 0.44 for the same comparison.

In the particular case of this work, the transport number decrease can be attributed to the fact that high concentrations of LiCl and LiOH promote higher concentration of Li^+^ in the membrane as a counterion. This, along with the principle of electroneutrality and Donnan exclusion, causes increase in the concentration of Cl^−^ and OH^−^ co-ions in the membrane [48]. According to the Nernst–Planck flux equation applied to the membrane, the latter would promote more OH^−^ ion leakage across the cation-exchange membrane due to increased concentration and current density [54,55]. In this work, this is manifested by a greater influence of the LiOH solution on the lithium transport number compared to the LiCl solution, which can be attributed to the presence of OH^−^ as a co-ion in the membrane.

Regarding the membranes used, the CMX membrane presents a higher transport number for lithium ions than the CMB membrane, which can be attributed to its lower thickness and electrical resistance. On the other hand, at high LiOH concentrations (8.0 wt%), an increase in current density increases the lithium transport number—especially for the CMX membrane.

### 3.3. Current–Voltage Curves

**Figure 7:**
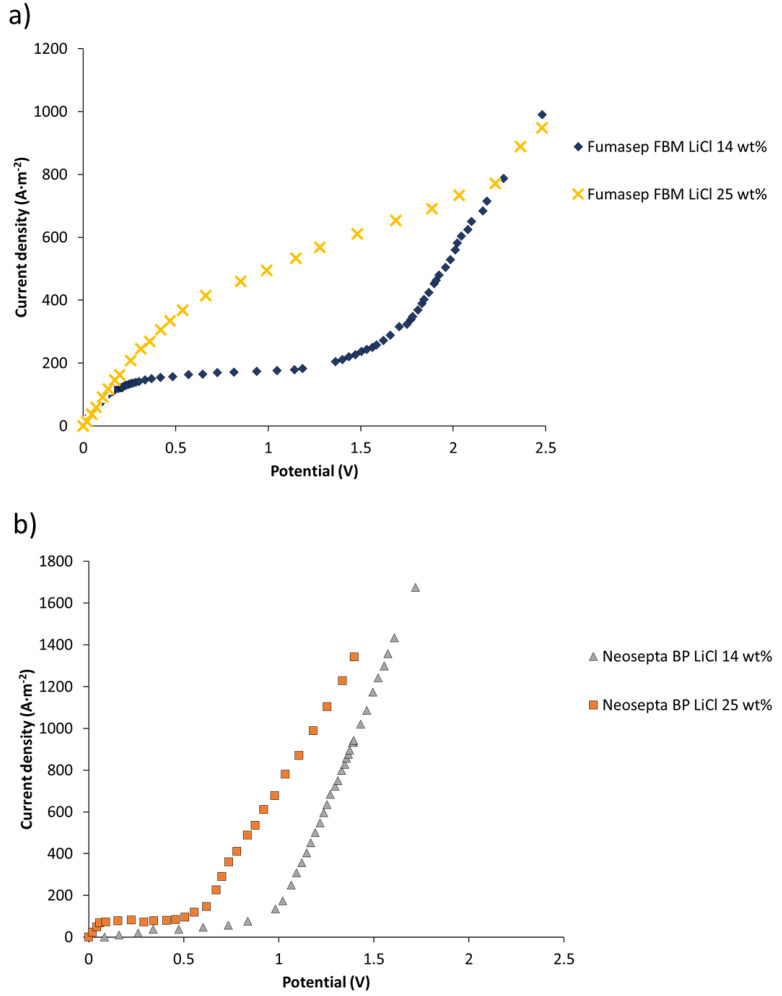
Salt leakage current–potential curves in bipolar membranes: (**a**) Fumasep FBM; (**b**) Neosepta BP.

**Figure 8:**
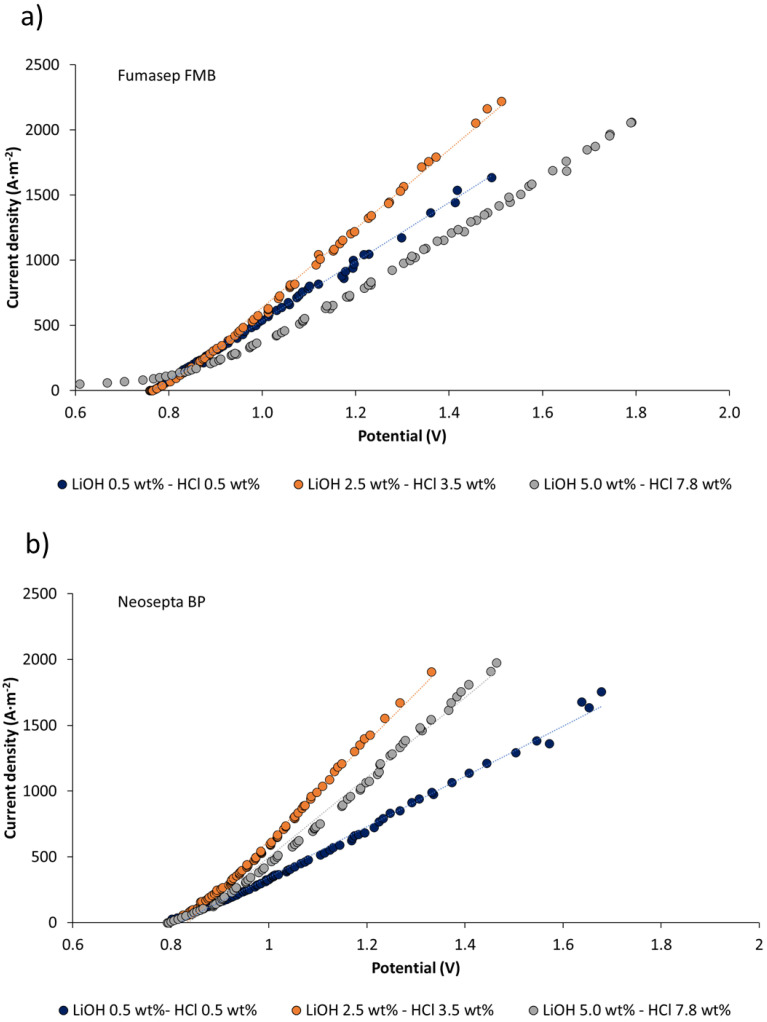
Linear sweep voltammetry of bipolar membranes under production operating conditions: (**a**) Fumasep FBM; (**b**) Neosepta BP.

**Figure 9:**
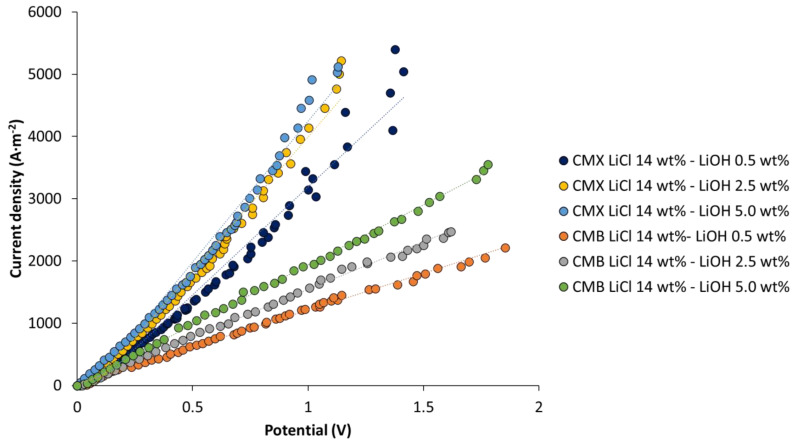
Linear sweep voltammetry in cationic membranes.

To determine the performance of bipolar and ion-exchange membranes, linear sweep voltammetry was performed as presented in Figure 7, Figure 8 and Figure 9.

#### 3.3.1. Salt Leakage through Bipolar Membranes

In the BMED process for LiOH production, Li^+^ leakage into the HCl solution and Cl^−^ leakage into the LiOH solution through the bipolar membrane can occur.

Figure 7 presents current–voltage curves measured for the determination of salt transport through bipolar membranes at two different concentrations: LiCl 14 wt% and 25 wt%. Results are presented for the Fumasep FBM and Neosepta BP membranes. At a concentration of LiCl 14 wt%, a plateau in the curve was clearly observed for both membranes, indicating a limiting current density associated with the transport of salts through the membrane. At 25 wt% LiCl concentration, the plateau was less defined and presented a slope, which is probably due to a significant transport of co-ions—which carry electric current—through the membrane [44]. When using 14 wt% LiCl as an electrolyte, the measured limiting current densities were 176 A∙m^−2^ and 77 A∙m^−2^ for the Fumasep FBM and Neosepta BP membranes, respectively. In contrast, for a 25 wt% LiCl electrolyte, the measured limiting current densities were approximately 334 A∙m^−2^ and 79 A∙m^−2^, respectively. Limiting current densities for the Neosepta BP membrane were lower, indicating that it possesses characteristics more suitable for operating at high electrolyte concentrations (see Table 2). Studies have been reported that indicate a decrease in salt leakage through bipolar membranes by increasing the thickness of one of the cationic or anionic layers that compose a bipolar membrane [44]. A similar effect can be expected when comparing the Neosepta BP and Fumasep FBM membranes, with the former being thicker.

For production process application, results indicate that for high concentrations (25% LiCl), the use of high current densities could reduce undesired transport of salts (for instance, in the case of the bipolar membrane Fumasep FBM, a current density of 1000 A∙m^−2^). Thus, electric current through bipolar membranes is transported mostly by H^+^ and OH^−^ ions generated by water dissociation in the bipolar membrane, and to a lower extent by leakage of Li^+^ and Cl^−^ salts. 

Balster et al. [44] studied salt leakage through a BP-1 membrane using the same characterization method. They found that the first limiting current density associated with salt transport was 0.61 mA∙cm^−2^ (6.1 A∙m^−2^) using a 2 M NaCl solution (approximately 11 wt%). By laminating AMX membranes on the anionic face of the BP-1 membrane (asymmetric bipolar membranes), they achieved a 25–30% decrease in the first current density, at the cost of an increase in electrical resistance by 32% and 84% by the addition of one and two AMX membranes, respectively. As reported in their study, the first limiting current density decreases with the thickness of the anionic layer, meaning reduced salt leakage through the bipolar membrane and, therefore, a base with fewer impurities might be obtained. 

In none of the obtained curves (see Figure 7) the second limiting current density—associated with the limited diffusion of water molecules towards the catalytic interface of the bipolar membrane—was observed.

#### 3.3.2. Linear Sweep Voltammetry on Bipolar Membranes under Production Conditions 

Figure 8 presents current–voltage curves for Fumasep FBM and Neosepta BP membranes at different degrees of LiOH and HCl concentration. The A/V ratio (amperes/voltage) has a linear behavior for measured potential range. When comparing the effects of LiOH and HCl concentration processes, for the same current density, the voltage drop tends to decrease when reaching a concentration of 2.5 wt% LiOH and 3.5 wt% HCl. Then, it presents a tendency to increase at higher concentrations (LiOH 5.0 wt% and HCl 7.8 wt%). For both types of membrane, the highest A/V value is obtained for 2.5 wt% LiOH and 3.5 wt% HCl concentrations. This means lower apparent electrical resistance of the membranes at such concentrations, which can be attributed to increased electrolytic conductivity in the solution on the membrane surface and osmotic deswelling at higher concentrations (LiOH 5.0 wt% and HCl 7.8 wt%), leading to an increase in membrane electrical resistance [56]. When LiOH and HCl concentration increases—up to 5.0 wt% and 7.8 wt%, respectively—A/V ratio decreases and apparent electrical resistance increases, suggesting that there is an optimal concentration value close to 2.5 wt% LiOH and 3.5 wt% HCl, where the membrane voltage drop is lower. Regarding the increase in the electrical resistance of the bipolar membrane at concentrations of 5.0 wt% LiOH and 7.8 wt% HCl, this can be attributed to concentrated electrolyte that is absorbed by the membrane [57]. At high concentrations, the effect of membrane dehydration has been observed [56,57]; at the same time, membrane conductivity decreases due to absorbed electrolyte by the membrane. At 5.0 wt% HCl and 7.8 wt% LiOH concentrations, the Fumasep FBM membrane exhibits higher apparent electrical resistance than the Neosepta BP membrane. This can be attributed to its higher electrolyte absorption compared to the Neosepta FBM membrane (see Table 6). The latter is associated with a lower electric charge density, which is inversely proportional to water uptake [58].

The dissociation voltage of water in bipolar membranes has been reported to be 0.83 V, but it is usually higher in practical applications [59,60]. In the case of linear sweep voltammetry in Figure 8, the dissociation voltage measured for the Fumasep FBM and Neosepta BP membranes was 0.76–0.80 V and 0.82–0.84 V, respectively. Low water dissociation voltages observed for the Fumasep FBM membrane have also been reported by Xu et al. [61], and can be attributed to an exergonic secondary neutralization reaction between the OH^−^ and H^+^ ions, which reduces the electrical energy requirement for water electrolysis. On the other hand, this result can be attributed to leakage current associated with a non-ideal selectivity of the membrane [62].

In the case of the Fumasep FBM membrane at higher concentrations (5.0 wt% LiOH and 7.8 wt% HCl), a small plateau is detected in the graph for potential values below 0.8 V. This can be explained by the increase in salt leakage with concentration. For practical purposes of the production of bases and acids at high concentrations, the bipolar membrane Neosepta BP presents better performance with respect to salt leakage reduction.

#### 3.3.3. Linear Sweep Voltammetry in Cation-Exchange Membranes

Linear sweep voltammetry was performed on the CMX and CMB membranes at different LiOH concentrations. Figure 9 presents a comparative plot of the different current–voltage curves obtained. Current variation with potential was linear for the measurement range used, and limiting current density was not reached in any case. It can be observed from the graph that current densities close to 5000 A∙m^−2^ for the CMX membrane, and between 2200 and 3500 A∙m^−2^ for the CMB membrane, were reached for a potential range between 0 and 1.8 V. This indicates that the high electrolyte concentrations studied allow high availability of Li^+^ ions on the membrane surface to migrate across the membrane without the occurrence of concentration polarization. The highest current densities were reached at higher electrolyte concentrations. For theCMX membrane, the current density at 5.0 wt% LiOH was on average 32% higher than the value at 0.5 wt% LiOH. In the case of the CMB membrane, this difference was 60%. On the other hand, when comparing the membranes at the same concentration, the CMX membrane allowed us to obtain current densities 113–159% higher than those obtained with the CMB membrane. Better energy efficiency was observed for the CMX membrane, as it achieved a higher electric current density at a lower potential difference. This can be attributed to the fact that the CMX membrane has 13–21% less thickness than the CMB membrane (see Table 7), and an electrical resistance 22–24% lower (see Table 1).

For the application of the membranes in LiOH production, the selected concentrations and flux rates (1.0–1.4 cm/s) were adequate for lithium transport through cationic membranes without reaching the limiting current density.

### 3.4. Long-Running Production Tests of LiOH by BMED

Long-running tests of LiOH production were performed using two different bipolar membranes (Neosepta BP and Fumasep FBM) and cation-exchange membranes (CMX and CMB) (Table 5). The current densities used were 500 and 1000 A∙m^−2^. These current densities were chosen according to the LSV results in order to reduce salt leakage through the bipolar membranes (see Figure 7). The initial LiCl feed concentration was between 14 and 34 wt%. Initial LiOH and HCl concentrations equal to 0.5 wt% were used in all tests. The obtained results are summarized in Table 8.

#### 3.4.1. Product Purity

**Figure 10:**
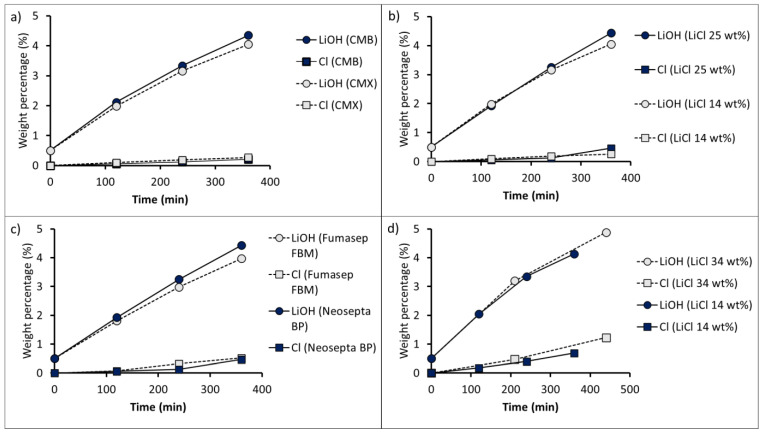
Concentration variation of LiOH and Cl^−^ in LiOH solution according to different operating conditions: (**a**) comparison of the cation-exchange membranes CMX and CMB (Test 1 and Test 2); (**b**) comparison of 14 and 25 wt% LiCl concentrations at 1000 A∙m^−2^ (Test 1 and Test 3); (**c**) comparison of the bipolar membranes Neosepta BP and Fumasep FBM (Test 3 and Test 4); (**d**) comparison of 14 and 34 wt% LiCl concentrations at 500 A∙m^−2^ (Test 5 and Test 6). Initial LiOH concentration 0.5 wt%.

Among the results, Figure 10 shows variation in LiOH and Cl^−^ ion concentrations over time according to different operating conditions. The presence of chloride as an impurity can be attributed to the leakage of Cl^−^ into the bipolar membrane and undesired transport of this anion across the cation-exchange membrane, due to high LiCl concentration and its effect on co-ion concentration in the membrane [48]. Cl^−^ molar flux into the LiOH compartment was calculated to be between 0.47 and 1.06 mol∙m^−2^∙h^−1^ when using a 14 wt% LiCl concentration, whereas for a 34 wt% LiCl concentration, Cl^−^ flux was between 1.29 and 2.28 mol∙m^−2^∙h^−1^, evidencing the influence of LiCl concentration on undesired Cl^−^ transport.

In relation to the two cation-exchange membranes used, the CMB membrane (Test 1) presented a LiOH concentration on average 6.6% higher than that obtained with the CMX membrane (Test 2), with a Cl^−^ content of 0.21 wt% and 0.26 wt%, respectively (see Figure 10a). This means that with the CMB membrane, as the LiOH solution was concentrated from 2.11 wt% to 4.35 wt%, the solution purity decreased from 97.9% to 95.4%, respectively. On the other hand, in the case of the CMX membrane, the increase in LiOH concentration from 1.98 wt% to 4.05 wt% implied a reduction in purity from 94.6 to 93.4%. The latter can be attributed to the fact that the CMX membrane is thinner and shows lower water uptake, presenting less resistance to undesired Cl^−^ diffusion and OH^−^ leakage through it.

Figure 10b presents the LiOH concentration results when using different LiCl concentrations (14 wt% and 25 wt%) at 1000 A∙m^−2^. The use of a 25 wt% LiCl concentration allowed us to obtain a final LiOH concentration 9.4% higher compared to the use of a 14 wt% LiCl solution. However, this implies a 76.9% higher Cl^−^ content (see Test 1 and 3 in Table 8). After 360 min of processing, the final LiOH purity was 93.4% and 88.6% when using 14 wt% and 25 wt% LiCl solutions, respectively.

Regarding the comparison of bipolar membranes in Tests 3 and 4 (see Figure 10c), a final LiOH concentration of 4.43 wt% and 3.97 wt%. with a Cl^−^ content of 0.52 wt% and 0.46 wt%, was obtained for the Neosepta BP and Fumasep FBM membranes, respectively. The LiOH solution purity decreased with LiOH concentration. When the Neosepta BP bipolar membrane was used (Test 3), the LiOH solution purity decreased from 96.0% to 88.6% when concentrated from 1.93 wt% to 4.43 wt%. On the other hand, in the case of the Fumasep FBM membrane (Test 4), the solution purity decreased from 92.9% to 83.8% when concentrating the LiOH solution from 1.81 wt% to 3.97 wt%.

In Tests 5 and 6, the use of four three-compartment cells at a current density of 500 A∙m^−2^ (see Figure 10d) resulted in a final LiOH solution with higher Cl^−^ content. This can be attributed to an increase in Cl^−^ diffusion into the cation-exchange membrane as the total membrane area in the stack increased, and the current density of 500 A∙m^−2^ was not able to mitigate bipolar membrane Cl^−^ ion leakage. When using a 14 wt% LiCl feed (Test 5), the LiOH solution purity decreased from 90.7% to 83.6% when concentrating from 2.05 wt% to 4.13 wt% LiOH. On the other hand, when a 34 wt% LiCl feed was used (Test 6), Figure 10d shows that, at 210 min of operation, LiOH concentration increased from 0.50 wt% to 3.10 wt%, with a Cl^−^ concentration of 0.47 wt%. After this point, an accelerating trend in the increase of Cl^−^ concentration in the LiOH compartment can be observed in the graph, reaching 1.23 wt% after 440 min (corresponding to a purity of 66.0% LiOH in solution). This behavior was accompanied with a decrease in LiOH concentration rate, as observed in a change of slope in the curve, suggesting a greater leakage of Cl^−^ ions through the bipolar membrane promoted by HCl concentration increase. On the other hand, there was a higher undesired transport of Cl^−^ ions across the cation-exchange membrane due to a high LiCl concentration (34 wt%), affecting the final product purity.

#### 3.4.2. SEC and Current Efficiency

**Figure 11:**
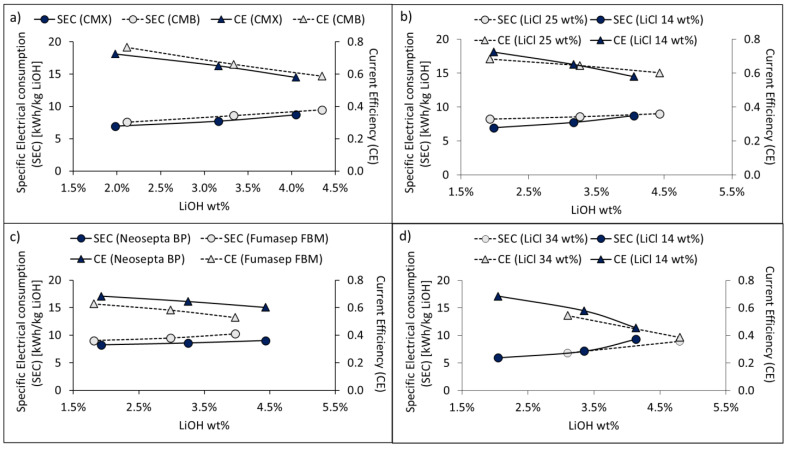
Specific electricity consumption and current efficiency under different operating conditions: (**a**) comparison of the cationic membranes CMX and CMB (Test 1 and Test 2); (**b**) comparison of 14 and 25 wt% LiCl concentrations at 1000 A∙m^−2^ (Test 1 and Test 3); (**c**) comparison of the bipolar membranes Neosepta BP and Fumasep FBM (Test 3 and Test 4); (**d**) comparison of 14 and 34 wt% LiCl concentrations at 500 A∙m^−2^ (Test 5 and Test 6) Initial LiOH concentration 0.5 wt%.

Figure 11 shows the total specific electricity consumption (SEC) and current efficiency (CE) corresponding to LiOH production in long-running tests.

Energy efficiency according to cation-exchange membrane type was compared based on the results of Tests 1 and 2, and the results are presented in Figure 11a. For Test 1 with the Neosepta BP bipolar membrane and the CMX membrane, when concentrating the LiOH solution from 1.98 wt% to 4.05 wt%, specific electricity consumption (SEC) increased by 25.6% and current efficiency (CE) decreased by 19.9%. On the other hand, in Test 2, when using the CMB membrane, SEC increased by 24.8% and CE decreased by 23.4%. However, the average SEC and CE with the CMB membrane were 10% and 3% higher, respectively, compared to the CMX membrane. The higher SEC obtained with the CMB membrane can be attributed to the higher electrical resistance of this membrane (see Table 1).

Figure 11b compares SEC and CE for the process at 1000 A∙m^−2^ for initial LiCl solutions of 14 wt% and 25 wt% according to Tests 1 and 3, respectively. For Test 1, with 14 wt% LiCl solution, when concentrating the LiOH solution from 1.98 wt% to 4.05 wt%, SEC increased by 25.6% and CE decreased by 19.9%. Meanwhile, for Test 3, with 25 wt% LiCl solution, when concentrating the LiOH solution from 1.93 wt% to 4.43 wt%, SEC increased by 9.5% and CE decreased by 11.8%. With 14 wt% LiCl solution, a 12.9% lower SEC and 3.6% higher CE was observed up to LiOH concentrations of 3.16–3.25 wt%. After this point, energy efficiency reduction could be attributed to a decrease in the concentration and electrical conductivity of the LiCl solution over time. When initial LiCl solutions of 14 wt% and 25 wt% were used, the final LiCl concentration was 8.71 wt% and 18.16 wt%, respectively.

The influence of the bipolar membranes Neosepta BP and Fumasep FBM influence can be observed when comparing Tests 3 and 4 in Figure 11c. When the Neosepta BP membrane was used, SEC was 10% lower compared to the Fumasep FBM membrane, while CE was 10% higher. The latter results in a better use of electric current in OH^−^ production by the Neosepta BP membrane, resulting in a final LiOH concentration of 4.43 wt%—11.6% higher when compared to the Fumasep FBM membrane. With the Neosepta BP membrane, when concentrating LiOH solution from 1.93 wt% to 4.43 wt%, SEC increased by 9.5% and CE decreased by 11.8%. On the other hand, when concentrating the LiOH solution from 1.81 wt% to 3.97 wt% with the Fumasep FBM membrane, SEC increased by 13.9% and CE decreased by 15.7%. These results show a higher energy yield when using the Neosepta BP membrane.

The effects of LiCl concentrations of 14 wt% (Test 5) and 34 wt% (Test 6) with four three-compartment cells and a current density of 500 A∙m^−2^ are presented in Figure 11d. For Test 5, with 14 wt% LiCl solution, when concentrating the LiOH solution from 2.05 wt% to 4.13 wt%, specific electricity consumption (SEC) increased from 5.97 to 9.29 kWh∙kg^−1^, while electrical current efficiency (CE) decreased from 0.69 to 0.46, respectively. On the other hand, for Test 6, Figure 11d shows that by concentrating LiOH from 0.50 wt% to 3.10 wt%, a specific electricity consumption of 6.81 kWh per kg LiOH was obtained, which then increased to 8.92 kWh∙kg^−1^ when reaching a LiOH concentration of 3.80 wt%. When using a 14 wt% LiCl solution (Test 5), current efficiency was on average 6.2% higher compared to 34 wt% LiCl solution (Test 6). Nevertheless, after reaching a LiOH concentration of 3.35 wt%, CE decreased in an accelerated way due to a decrease in LiCl concentration.

For a LiCl concentration of 14 wt%, specific electricity consumption was between 12% and 20% lower compared to 34 wt% LiCl. This can be attributed to a decrease in electrolytic conductivity in concentrated aqueous LiCl solutions greater than 7 mol∙kg^−1^ [63] (approximately 23 wt%).

The lowest SEC value obtained was 5.97 kWh per LiOH kg in Test 5 after the first 120 min of processing, when reaching a LiOH concentration of 2.05 wt%. This can be attributed to the lower current density used (500 A∙m^−2^) and the advantage of increasing the number of membranes in the stack to four LiOH compartments. However, current efficiency (CE) when concentrating the LiOH solution to 3.35 wt% was 0.58, and as LiOH concentration increased to 4.13 wt%, the current efficiency decreased to 0.46. Thus, part of the electric current was consumed in leakage by Cl^−^ migration across the bipolar membrane, rather than in OH^−^ production. This is consistent with the results obtained in the LSV tests. This behavior evidences the advantage of using operational current densities higher than the first limiting current density in bipolar membranes.

According to our results, process energy efficiency is affected with increasing LiOH concentration according to OH^−^ leakage effects and cation membrane deswelling. On the other hand, increasing HCl solution concentration promotes a higher leakage of Cl^−^ ions into the LiOH compartment, affecting current efficiency in the bipolar membrane in the generation of OH^−^ ions, which also affects solution purity.

Table 8 presents a summary of results obtained in long-running tests. The best result was achieved in Test 2, where a Neosepta BP membrane, CMB cationic membrane and current density of 1000 A∙m^−2^ were used. The possibility was shown of achieving LiOH solutions between 3.34 and 4.35 wt% concentration, with a range of 96.0–95.4% purity. Product purity is therefore inversely proportional to the final concentration obtained. The lowest average specific electricity consumption (SEC) was achieved in Test 1 between 6.94 and 8.71 kWh per kilogram of LiOH. On the other hand, the highest electrical current efficiency (CE) was achieved in Test 2, in the range of 0.77–0.59, with an SEC between 7.57 and 9.45 kWh per kilogram of LiOH.

The CMB membrane presented a high current efficiency despite its higher electrical resistance, which can be attributed to a higher resistance to co-ion leakage. However, it is not recommended to use it with LiCl solutions higher than 14 wt%. This membrane was tested in long-running tests at 25 wt% LiCl concentrations, showing damage to its structure after two hours of processing due to the high osmotic pressure reached, causing its rupture and contamination between LiCl and LiOH solutions. Consequently, these results are not presented. 

#### 3.4.3. Specific Electricity Consumption (SEC) Comparison

Specific BMED electricity consumption depends mainly on membranes’ characteristics and their interaction with concentrated solutions under a specific electric current density. The SEC results obtained in this work were determined by final LiOH concentration, and are comparable to other membrane processes used for lithium recovery and LiOH production. Specific energy consumptions between 5.43 and 6.20 kWh∙kg^−1^ of LiOH from lithium-rich solutions produced from salt lake brine have been reported for processes such as nanofiltration, reverse osmosis, and conventional electrodialysis integrated with BMED, achieving final LiOH concentrations between 0.58 and 1.03 M (approximately 1.37–2.41 wt%), with current efficiencies between 0.36 and 0.44% [64]. Regarding direct BMED application, SEC in the order of 6.60 kWh∙kg^−1^ was reported from 0.725–0.730 wt% LiCl solutions containing organic solvents reaching 0.3 M LiOH concentrations (approximately 0.71 wt%) [28], while recently, from LiCl solutions of 100 g∙L^−1^ (approximately 9.5 wt%), SECs between 2.78 kWh∙kg^−1^ and 7.80 kWh∙kg^−1^ of LiOH have been reported, with current efficiencies between 0.7384 and 0.1445 at 640 A∙m^−2^ using CMB and BP membranes, achieving LiOH concentrations of 1.08 M and 2.40 M (approximately 2.5 wt% and 5.4 wt% LiOH), respectively [64]. These reports are consistent with the results presented in this work, demonstrating the influence of LiCl concentration and the degree of final LiOH concentration on process performance. 

The same technology has been used with different starting solutions. Thus, a 0.5 M Li_2_SO_4_ feed [65] reports a specific electricity consumption of 7 kWh∙kg^−1^, which increases when working with more concentrated solutions. Meanwhile, the use of aqueous Li_2_CO_3_ solutions up to 0.18 M presents an SEC of up to 20.4 kWh∙kg^−1^ of LiOH [31], depending on operating conditions.

On the other hand, LiOH production by membrane electrolysis has reported an SEC of 6.1–14.6 kWh∙kg^−1^ LiOH using initial LiOH solutions between 4 and 8 wt% as the initial catholyte [66] and 7.25 kWh∙kg^−1^ of LiOH for an initial catholyte of 2.3 wt% [29].

For conventional industrial process of obtaining lithium hydroxide from Li_2_CO_3_ by chemical reaction with lime slurry, the specific energy consumption is 14.04 kWh∙kg^−1^ of LiOH∙H_2_O (or 24.6 kWh∙kg^−1^ of LiOH) [67], of which electricity, fuel, and natural gas consumption represent 17.3%, 10.3%, and 72.4% of the total energy required, respectively. High natural gas consumption can be attributed to the thermal energy required for the evaporation and crystallization stages of the process to obtain lithium hydroxide monohydrate, in which a lithium hydroxide solution of approximately 3 wt% must be evaporated to saturation. 

The BMED process of obtaining LiOH does not eliminate the need to evaporate LiOH solution to crystallize LiOH∙H_2_O; however, it is expected that obtaining LiOH concentrations higher than 3 wt% would contribute to reduce heat requirements in these stages. Thus, if the LiOH solution is evaporated to saturation at 70°C, initial LiOH concentrations of 4 wt% and 5 wt% would allow the thermal energy requirement to be reduced by 9.4% and 18.9%, respectively, compared to using a 3 wt% LiOH solution. 

Obtaining LiOH via the chemical reaction of Li_2_CO_3_ with lime slurry presents a conversion efficiency of 59.0–59.5% after one hour of processing at 60–100 °C [68], which, based on mass and energy balances, allows the estimation of a theoretical SEC of 1.27–2.84 kWh∙kg^−1^ of LiOH. This required energy is lower than that obtained in this work by BMED. However, membrane processes could become competitive if process sustainability is considered by reducing the use of chemical reagents, reducing waste generation, and potential coupling with non-conventional renewable energies.

## 4. Future Challenges

The results in this work present the current scope of obtaining high concentrations of LiOH by BMED, variation of electrical energy consumption with concentration, and salt leakage related to Cl^−^ ion contamination of LiOH solution. The results of this work suggest that from an initial LiOH concentration of 0.5 wt%, it is possible to obtain concentrated LiOH solutions in the range of 3.34–4.35 wt%, with 96.0–95.4% purity and a specific electricity consumption between 7.57 and 9.45 kWh per kilogram of LiOH. After this point, current efficiency tends to decrease below 0.50, significantly increasing the specific energy consumption of the process. This is associated with high OH^−^ ion leakage in the cation-exchange membrane [40,53] and salt leakage in the bipolar membrane [44,69], which causes undesired Cl^−^ transport into the LiOH compartment. In the production of LiOH by membrane electrolysis, energy consumption can be reduced in a concentration range between 40 and 50 g∙L^−1^ [30], which is approximately 3.5 wt% and4.3 wt% LiOH, respectively. 

For the implementation of a lithium hydroxide production process by electrodialysis with bipolar membranes, there exist limitations related to membrane performance, affecting final product purity and energy efficiency. For the use of lithium hydroxide as a precursor for lithium batteries, work must be done to reduce the transport of impurities in membranes. Both monopolar and bipolar membranes are not 100% permselective. Thus, as LiOH and HCl concentrations increase, LiOH solution purity and process efficiency are affected.

Implementation-related challenges for this technology in LiOH production involve aspects such as the performance of monopolar membranes and bipolar membranes, stack design related to the optimal number of unit cells, membrane lifetime, and technology implementation coupled with renewable energy sources. 

In this work, evidence is shown that process efficiency is mainly established by membrane performance. To date, several investigations have been carried out in order to improve cationic membranes’ selective transport [70,71,72]. In the case of bipolar membranes, the salt leakage problem can be addressed by using asymmetric bipolar membranes [44]. However, this improvement in salt leakage reduction leads to higher electrical resistance and, therefore, an increase in electrical power consumption. Based on these investigations, it could be expected that new membranes will emerge in the future, being more suitable for application in the production of concentrated bases. Thus, the final LiOH concentration will be increased with monopolar membranes by higher resistance to OH^−^ leakage, and with bipolar membranes by reduced salt leakage and higher water dissociation efficiency.

Currently, these difficulties could be addressed by specific operating conditions. For instance, it has been shown in this work that at 14 wt% LiCl concentration, lower specific electricity consumption and higher electric current efficiency are obtained for LiOH production, so if a constant LiCl concentration could be established during LiOH production, this would increase energy efficiency, and would also help to slow down reduction in concentration difference between LiCl and LiOH solutions separated by the cationic membrane which, in turn, would contribute to reducing the rate of lithium transport decrease through this membrane [47]. On the other hand, with respect to Cl^−^ leakage in the bipolar membrane, an increase in Cl^−^ leakage rate related to simultaneous concentration of LiOH and HCl was observed. Given the molar mass difference between these salts, the mass concentration rate (wt%) of HCl solution is always higher compared to LiOH. Therefore, for production processes, a low HCl concentration can be set while LiOH concentration increases. In this way, Cl^−^ leakage into the LiOH compartment through the bipolar membrane would be reduced.

On the other hand, the application of a high current density would reduce the membrane area requirement for a given production, reducing the overall process cost, as membranes are expensive. Moreover, according to linear sweep voltammetry analysis and long-running tests, working at high current densities favors water dissociation over salt leakage [69]. However, current efficiency decreases and, therefore, specific energy consumption increases, in addition to the fact that catalytic interlayer may deteriorate, which would imply a decrease in membrane lifetime and an increase in voltage drop after a long period of operation [73]. 

In industry, obtaining a high concentration in the final LiOH solution (greater than 3.0 wt%) would bring benefits to the current crystallization process of LiOH∙H_2_O production, since it would reduce the current difference between LiOH concentration and saturation concentration at which crystallization of lithium hydroxide monohydrate begins. In addition, this would encourage the use of green technologies, given the potential use of this technology driven by renewable energy sources. The Atacama Desert in Chile is characterized by high levels of solar radiation—between 6.7 and 10.55 kWh∙m^−2^ per day [74,75]. The application of membrane technologies for LiOH production—such as membrane electrodialysis [29] and bipolar electrodialysis membranes—is a potential alternative to the LiCl to Li_2_CO_3_ and subsequent Li_2_CO_3_ to LiOH conversion steps. Obtaining Li_2_CO_3_ from lithium brine reports an approximate SEC of 1.31 kWh∙kg^−1^ of Li_2_CO_3_ [67], which implies an equivalent SEC of 6.83 kWh∙kg^−1^ of LiOH (considering obtaining 0.38 kg of LiOH per kg of Li_2_CO_3_ for a 59% reaction conversion [68]). Therefore, the authors estimate that specific electricity consumptions determined by BMED and other membrane technologies offset those obtained by conventional methods, with the advantage of being potentially driven by clean technologies given the conditions of the Atacama Desert. Future studies should be conducted to quantify the real impact of integrating solar energy into these processes.

The design choice of a high-purity lithium hydroxide production process would be conditioned by investment costs and operating costs. Pilot tests and process simulations would allow the obtaining of key information according to different scenarios and process configurations. The latter constitutes work in progress that the authors would like to present in a follow-up to the present results.

## 5. Conclusions

Experimental tests were developed to determine and analyze the scope and feasibility of BMED application in high-purity lithium hydroxide production. Among the main results, the influence of high concentrations of LiOH and LiCl solutions on LiOH production energy efficiency and final solution purity were determined and analyzed. The best results for energy efficiency were obtained at low initial LiOH concentrations. A high LiCl concentration (25–34 wt%) was shown to increase electrical resistance and promote Cl^−^ diffusion into the LiOH solution. On the other hand, increasing the LiOH concentration was shown to cause OH^−^ ion leakage phenomena in the cation-exchange membrane, reducing its performance. Regarding bipolar membranes, high LiOH and HCl concentrations were associated with accelerated leakage of co-ions, impairing LiOH solution purity. This can currently be addressed with asymmetric bipolar membranes, or by avoiding high HCl concentrations with a high current density that promotes water dissociation over salt leakage.

For the application of electrodialysis with bipolar membranes, the results of this work show that it is possible to obtain a LiOH concentration in a concentration range of 3.34–4.35 wt%, with 96.0–95.4% purity. After this point, electrical power consumption and LiOH contamination with chloride ions tend to increase significantly, affecting solution purity. From a 0.5 wt% LiOH solution and a current density of 1000 A∙m^−2^, a specific electrical energy consumption (SEC) of 9.45 kWh∙kg^−1^ was determined with a current efficiency (CE) of 0.77–0.59, obtaining a final LiOH concentration of 4.35 wt%. On the other hand, with a current density of 500 A∙m^−2^, an SEC of 5.97 kWh∙kg^−1^ was obtained with a CE of 0.69, obtaining a LiOH concentration of 2.05 wt%. The highest current efficiency obtained was 0.77 at 0.5 wt% LiOH and 14 wt% LiCl concentrations.

To achieve high LiOH concentrations with higher efficiency, it is necessary to improve the performance of bipolar membranes at high concentrations, and the resistance of cation-exchange membranes to OH^−^ leakage, supported by suitable operating conditions.

## Figures and Tables

**Table 1:** Monopolar membranes’ technical specifications [38,39,40,41].

Characteristics	AMX	CMX	CMB
Type	Anion	Cation	Cation
Electrical resistance (Ω∙cm^2^)	2.0–3.5 (0.5 M NaCl)	2.5–3.5 (0.5 M NaCl)	4.5
Thickness of wet membrane (mm)	0.14–0.18	0.17–0.19	0.21
Burst pressure	4.5–5.5 (kg/cm^2^)	5–6 (kg/cm^2^)	≥0.40 MPa
Exchange capacity (meq∙g^−1^)	1.4–1.7	1.5–1.8	2.4–2.7
Water content (%)	25–30	25–30	37–42
Thermal stability (°C)	40	40	60

**Table 2:** Bipolar membranes’ technical specifications.

Characteristics	Fumasep FBM [42,43]	Neosepta BP [27,38]
Type	Bipolar	Bipolar
Thickness (mm)	0.18–0.20 (wet) 0.13–0.16 (dry)	0.22 (wet)
Burst pressure	-	>0.40 MPa
Thermal stability (°C)	40	-
Water dissociation voltage at 100 mA∙cm^−2^	<1.2 ^1^	1.2 ^2^
Water dissociation efficiency at 100 mA∙cm^−2^	>98%	>98%

^1^ At 100 mA∙cm^−2^ in 0.5 M NaCl at 25 °C; ^2^ Potential difference measured between silver/silver chloride electrodes. 1 N NaOH/1 N HCl 10 A∙dm^−2^ 30 °C.

**Table 3:** Initial LiCl and LiOH concentrations in solution for lithium ion transport number determination.

Configuration	1	2	3	4	5	6
LiCl (wt%)	14	14	14	14	25	25
LiOH (wt%)	0.5	2.5	5.0	8.0	0.5	5.0

**Table 4:** Solution concentrations and membranes used in LSV tests.

**Configuration (a): Bipolar Membrane’s Salt Leakage**						
Test	1		2	3		4
Bipolar membrane	Fumasep FBM		Neosepta BP	Fumasep FBM		Neosepta BP
LiCl solution (wt%)	14		14	25		25
**Configuration (b): Bipolar Membrane’s Apparent Electrical Resistance**						
Test	1	2	3	4	5	6
Bipolar membrane	Fumasep FBM	Fumasep FBM	Fumasep FBM	Neosepta BP	Neosepta BP	Neosepta BP
LiOH solution (wt%)	0.5	2.5	5.0	0.5	2.5	5.0
HCl solution (wt%)	0.5	3.5	7.8	0.5	3.5	7.8
**Configuration (c): Cation-Exchange Membrane’s Apparent Electrical Resistance**						
Test	1	2	3	4	5	6
Membrane	CMX	CMX	CMX	CMB	CMB	CMB
LiCl solution (wt%)	14	14	14	14	14	14
LiOH solution (wt%)	0.5	2.5	5.0	0.5	2.5	5.0

**Table 5:** Operating conditions of long-running tests of LiOH production according to different bipolar membranes, initial LiCl concentration, and current density.

Test	1	2	3	4	5	6
Cation membrane	CMX	CMB	CMX	CMX	CMX	CMX
Bipolar membrane	Neosepta BP	Neosepta BP	Neosepta BP	Fumasep FBM	Fumasep FBM	Fumasep FBM
Current density (A∙m^−2^)	1000	1000	1000	1000	500	500
LiCl initial concentration (wt%)	14	14	25	25	14	34
Time (min)	360	360	360	360	360	440
Number of three-compartment cells	2	2	2	2	4	4

**Table 6:** Water uptake measured in cation-exchange and bipolar membranes.

Water Uptake (%)									
Solution	HCl 0.5 wt%	HCl 2.5 wt%	HCl 5.0 wt%	LiOH 0.5 wt%	LiOH 2.5 wt%	LiOH 5.0 wt%	LiCl 14 wt%	LiCl 25 wt%	LiCl 34 wt%
CMX	-	-	-	31.2 ± 0.6	30.5 ± 0.4	29.5 ± 0.2	27.1 ± 0.0	20.3 ± 0.6	13.8 ± 0.2
CMB	-	-	-	35.9 ± 0.7	38.0 ± 0.8	37.1 ± 0.8	30.6 ± 0.5	26.5 ± 0.8	18.9 ± 0.7
Fumasep FBM	37.5 ± 0.3	48.2 ± 0.2	40.6 ± 0.3	52.1 ± 0.6	55.2 ± 0.0	53.1 ± 0.0	-	-	-
Neosepta BP	31.1 ± 0.7	30.9 ± 0.3	30.4 ± 0.1	33.8 ± 0.4	35.9 ± 0.2	30.4 ± 0.6	-	-	-

**Table 7:** Wet membrane thickness measured in cation-exchange and bipolar membranes.

Thickness of Wet Membrane (μm)									
Solution	HCl 0.5 wt%	HCl 2.5 wt%	HCl 5.0 wt%	LiOH 0.5 wt%	LiOH 2.5 wt%	LiOH 5.0 wt%	LiCl 14 wt%	LiCl 25 wt%	LiCl 34 wt%
CMX	-	-	-	172 ± 1	170 ± 1	168 ± 1	167 ± 1	165 ± 1	164 ± 1
CMB	-	-	-	198 ± 2	198 ± 2	198 ± 1	196 ± 0	196 ± 1	195 ± 1
Fumasep FBM	176 ± 2	171 ± 1	171 ± 1	189 ± 1	191 ± 2	192 ± 1	-	-	-
Neosepta BP	229 ± 2	229 ± 1	230 ± 1	263 ± 1	266 ± 1	265 ± 2	-	-	-

**Table 8:** Summary of the main results obtained in long-running tests.

Test	Test 1			Test 2			Test 3			Test 4			Test 5			Test 6		
Cation membrane	CMX			CMB			CMX			CMX			CMX			CMX		
Bipolar membrane	Neosepta BP			Neosepta BP			Neosepta BP			Fumasep FBM			Fumasep FBM			Fumasep FBM		
Current density (A∙m^−2^)	1000			1000			1000			1000			500			500		
LiCl initial concentration (wt%)	14			14			25			25			14			34		
Number of unit cells	2			2			2			2			4			4		
LiOH concentration (wt%)	1.98	3.16	4.05	2.11	3.34	4.35	1.93	3.25	4.43	1.81	2.98	3.97	2.05	3.35	4.13	3.10	4.80	5.20
Cl^−^ concentration in LiOH solution (wt%)	0.10	0.19	0.26	0.05	0.13	0.21	0.06	0.13	0.46	0.07	0.32	0.52	0.18	0.40	0.69	0.48	1.23	1.24
Final purity (wt%)	94.6	93.5	93.4	97.9	96.0	95.4	96.0	95.3	88.6	92.9	86.5	83.8	90.7	87.8	83.6	76.8	66.0	54.7
SEC (kWh∙kg^−1^)	6.94	7.72	8.71	7.57	8.58	9.45	8.23	8.58	9.01	8.98	9.46	10.23	5.97	7.14	9.29	6.81	8.92	11.94
CE	0.72	0.65	0.58	0.77	0.66	0.59	0.68	0.64	0.60	0.63	0.58	0.53	0.69	0.58	0.46	0.54	0.39	0.31

## Data Availability

Not applicable.
